# Behavioral Intentions and Factors Influencing Nurses' Care of COVID-19 Patients: A Cross-Sectional Study

**DOI:** 10.3389/fpubh.2022.914599

**Published:** 2022-06-29

**Authors:** Feifei Cui, Yundan Jin, Haiying Wu, Rongting Wang, Xinling Pan, Shuainan Chen, Yanyan Jin, Meiqi Yao, Huiqiang Fan, Jing Xu

**Affiliations:** ^1^Department of Nursing, The Affiliated Dongyang Hospital, Wenzhou Medical University, Dongyang, China; ^2^Department of Biomedical Sciences Laboratory, The Affiliated Dongyang Hospital, Wenzhou Medical University, Dongyang, China; ^3^Department of Nursing, School of Medicine, The Second Affiliated Hospital, Zhejiang University, Hangzhou, China; ^4^Department of Nursing, Foresea Life Insurance Xi'an Hospital, Xi'an, China

**Keywords:** COVID-19, behavior intentions, self-efficacy, ethical cognition, influencing factors

## Abstract

**Objective:**

Behavioral intentions to care for patients with infectious diseases are crucial for improving quality of care. However, there have been few studies of the behavioral intentions and factors influencing patient care by clinical nurses during the COVID-19 pandemic. This study aims to explore cognition, attitudes, subjective norms, self-efficacy, and behavioral intentions of clinical nurses while caring for COVID-19 patients and to explore any influencing factors.

**Method:**

A cross-sectional survey was conducted of nurses through convenience sampling in southeast China from February 2020 to March 2020. The questionnaire was developed based on the theory of planned behavior and self-efficacy.

**Results:**

A total of 774 nurses completed the survey. Of these, 69.12% (535/774) reported positive behavioral intentions, 75.58% (585/774) reported a positive attitude, and 63.82% (494/774) reported having the confidence to care for patients. However, the lack of support from family and friends and special allowance affected their self-confidence. Attitude, self-efficacy, subjective norms, and ethical cognition were significantly positively correlated with behavioral intentions (r = 0.719, 0.690, 0.603, and 0.546, respectively, all *P* < 0.001). Structural equation model showed that self-efficacy, attitude, ethical cognition, and subjective norms had positive effects on behavioral intentions (β = 0.402, 0.382, 0.091, and 0.066, respectively, *P* < 0.01). The total effect of behavioral intentions was influenced by attitude, ethical cognition, self-efficacy, and subjective norms (β = 0.656, 0.630, 0.402, and 0.157, respectively, *P* < 0.01). In addition, ethical cognition had a positive mediating effect on behavioral intentions (β = 0.539, *P* < 0.001).

**Conclusion:**

The study results indicated that attitude, ethical cognition, and self-efficacy were the main factors influencing nurses' behavioral intention. Efforts should be made to improve nurses' attitude and self-efficacy through ethical education and training to increase behavioral intentions to care for patients with infectious diseases, which will improve the quality of nursing care.

## Introduction

The novel coronavirus disease 2019 (COVID-19) has been declared a pandemic by the World Health Organization and continues to spread globally, posing an unprecedented threat to public health (1). This large-scale public health challenge has resulted in fear and psychological stress among front-line nurses who are impacted by the unknown future of the disease and its highly contagious transmission and closed management (1–4). Previous studies have found that most nursing staff caring for patients with infectious diseases agree that patients should receive appropriate medical care and that nurses should continue to perform their responsibilities; however, they are worried about the health of themselves and their families and they are hesitant about personally participating in the care of patients with infectious diseases (5–8). The perception of occupational exposure may lead to an unwillingness to go to work, especially when they witness their colleagues acquire the disease. For fear of acquiring the virus, nurses may hesitate to provide the usual care or they minimize their caring hours (9). A phenomenological study found that dealing with COVID-19 patients for the first time was associated with fear, tension, anxiety, and worries. Most of the participants expressed a fear feeling of being infected or spreading the infection to their family (10). One study showed that work-environment risk category were found to have a significant positive relationship with the nurses' willingness to care for patients with COVID-19, which is a strong predictor for nurses' willingness (9). Adaptation susceptibility, threat prediction, fear, understanding of coronavirus disease, the occurrence of workplace death, understanding of disease transmission and casualty, negative beliefs, work pressure, social pressure, normative beliefs, and subjective norms have been identified as factors influencing nurses' care of infectious patients (11). Nurses with a higher level of knowledge were more willing to work with COVID-19 patients (10). A phenomenological study revealed that lack of knowledge about COVID-19 increased their anxiety and stress (9). So when nursing staff care for patients with infectious diseases, their understanding of the disease, ethical cognition, psychological preparation, behavior, attitude, and willingness are closely related to the quality of care (5, 12–14).

As nurses play an important role in providing therapeutic measures, understanding nurses' beliefs from a theoretical framework is important for updating intervention strategies and encouraging better nursing intention behavior (11). The theory of planned behavior (TPB) holds that behavioral attitudes, subjective norms, and perceived behavioral control determine behavioral intentions, which then predicts actual actions through behavioral intentions (15). TPB can be used to predict behavioral intentions, individual subjective normative attitudes, and self-efficacy of caring for patients with infectious diseases (16).

Clarifying nurses' willingness to care for COVID-19 patients may be critical to improving the quality of care. Hence, this study aimed to explore the behavioral intentions and influencing factors of nurses caring for COVID-19 patients. The findings may provide some insights for clinical nursing occupational protection education and nursing managers to formulate corresponding countermeasures.

## Methods

### Study Architecture, Study Sample, and Inclusion Criteria

**Figure 1:**
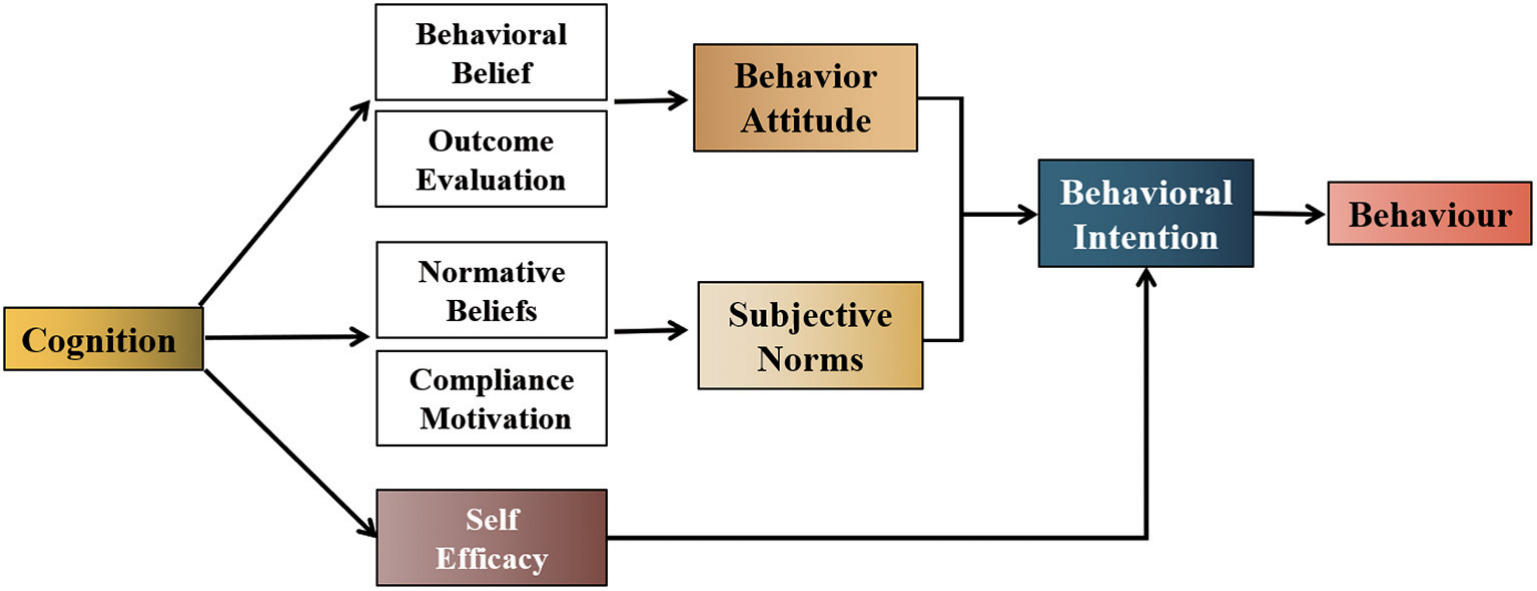
Architecture of behavioral intentions based on planned behavior, self-efficacy, and knowledge-attitude-practice theory. Definitions: Cognition: understanding of a disease, nurses' responsibility to care for patients, and nurses' rights and responsibilities to participate in care. Behavioral beliefs: a person's judgment of the likelihood of outcomes resulting from engaging in patient care. Outcome evaluation: a person's positive or negative value judgment of outcomes resulting from engaging in patient care. Normative belief: an individual's belief that he or she should engage in caring for patients with respect to important reference objects. Compliance motivation: the willingness of individuals to comply with important reference objects as far as patients are concerned. Behavior attitude: the positive or negative evaluation of an individual's behavior that reflects their preference for the behavior. Subjective norms: social pressures perceived by individuals while performing behaviors that reflect the influence of important others or groups. Self-efficacy: self-assessment of ability and confidence to care for a patient in a particular situation. Behavioral intentions: a person's subjective probability or preference for engaging in the care of patients.

The framework of this study, which is based on a literature review, panel discussions, and the theories of planned behavior and self-efficacy, and the research objectives are presented in Figure 1.

A descriptive cross-sectional survey was conducted by convenience sampling of nurses in southeast China from February 2020 to March 2020. Given the travel restrictions and enforcement of social distancing, web-based digital data collection has been accepted as an effective way to gain insights into individuals' behavioral intention during pandemics. To collect data, the Sojump online questionnaire platform (a secure online data collection survey tool that allows participants to answer questions conveniently and anonymously) was implemented and the link of questionnaire was shared through WeChat social software. Participants were briefed about the study purpose through written information reported on the questionnaire's homepage. Informed consent was obtained from all nurses before filling out the online questionnaire. Privacy was assured because no IP address was registered and no sensitive data were requested. In order to obtain adequate samples and ensure quality, the survey was conducted with the assistance of the Nursing Institute. The inclusion criteria were as follows: (1) nurses with a practicing qualification certificate and (2) clinical nurses with ≥1 year work experience. Non-clinical front-line nurses were excluded. This study was approved by the Clinical Research Ethics Committee of the Affiliated Dongyang Hospital, Wenzhou Medical University (Approval No. 2022-YX-085).

## Measures

### Questionnaire Construction

The questionnaire was designed based on planned behavior, self-efficacy and knowledge, and belief and action theory combined with a literature review, qualitative interviews, and two rounds of expert group discussion and included three parts. The first part assessed participants' demographic characteristics including age, gender, professional title, educational attainment, marital status, number of children, children's education, length of nursing career, care experience, and other contents. The second part was the ethical cognition questionnaire for caring for patients with infectious diseases, which was used to evaluate the ethical cognition of nurses. The questionnaire contained five items regarding a patient's right to receive care. Nurses' responses were measured on a 5-point Likert scale ranging from 1 = “strongly disagree” to 5 = “strongly agree”. The total scores were calculated by summing the scores of all five items and ranged from 5 to 25, with higher scores indicating greater positive cognition and attitudes about caring for patients with infectious diseases. The third part was a self-designed behavioral intention questionnaire used to assess nurses' willingness to care for COVID-19 patients. The questionnaire consisted of 29 items including 4 items on behavioral attitude, 4 items on subjective norms, 6 items on normative beliefs, 6 items on compliance motivation, 6 items on self-efficacy, and 3 items on behavioral intention. Responses were recorded using a 5-point Likert scale ranging from 1 = “strongly disagree” to 5 = “strongly agree”. Scores of 3–4 were positive while scores of 1–2 were negative, such that higher scores indicate stronger behavioral intentions.

### Questionnaire Validity Test

The content validity of the questionnaire was evaluated by twelve clinical senior nurses with experience in crisis management related to SARS, COVID-19 or other infectious diseases prevention and management. For the overall questionnaire, the content validity index was 0.962. Cronbach's alpha coefficients of internal consistency of ethical cognition and behavioral intention questionnaire were 0.697 and 0.884, respectively, and the retest reliability coefficients were 0.757 and 0.928, respectively.

A preliminary survey was conducted among 15 nursing staff who participated in the care of COVID-19 patients. During the formal questionnaire survey, 15 nurses were resurveyed and the results were analyzed. Correlation analysis revealed that the coefficients between the scores of each item and the total questionnaire score were 0.307–0.973 (*P* < 0.01). All of the correlation coefficients were >0.30, indicating that the items had good identification ability and discrimination.

Exploratory factor analysis was used to evaluate the structural validity of the questionnaire. The results showed a (Kaiser Meyer Olkin, KMO) value of 0.957 and a Bartlett's sphericity test value of 55,249.316 (df = 2016, *P* < 0.001). Principal component analysis (PCA) was used to perform skew rotation of factors using the standard of an eigenvalue ≥ 1. The results showed that the overall cumulative variance explanation rate was 71.25%, the total variance explanation rate of the corresponding dimensions of each item was >60%, and the factor load of each item was 0.410–0.961, indicating that the questionnaire had good structural validity.

### Statistical Analysis

Statistical analysis was performed using SPSS version 20.0 and AMOS version 24.0 (IBM, Chicago, IL, USA). Normally distributed continuous variables are reported using the mean ± standard deviation (SD). For non-normally distributed data, continuous variables are reported as the median with the interquartile range (25th and 75th percentiles). Categorical data are reported as *n* (%). Pearson correlation was used to analyze the relationship between social-demographic characteristics, attitude, self-efficacy, subjective norm, ethical cognition, and behavioral intentions. Multiple linear regression analysis was used to conduct multi-factor analysis. Structural equation modeling (SEM) was used to verify and analyze the logical relationship and mechanism among cognition, intention and attitude, subjective norms, and self-efficacy. A *P*-value < 0.05 was considered to be statistically significant.

## Results

### Participant Characteristics

A total of 836 nurses were recruited for this study, of which 774 (92.58%) provided valid responses included in the analysis. The demographic characteristics of the respondents are presented in Table 1. Of the total participants, 755 (97.55%) were female and 60.47% were aged 21–30 years old. The mean length of nursing career was 9.30 years (SD = 6.15, range: 1–40 years). A total of 295 (38.11%) participants were senior nurse and 65.76% had a bachelor's degree or above. More than half of participants were married (*n* = 458, 59.17%). Only 8.01% of participants had experience caring for patients with SARS or influenza A (H1N1), and more than 95% of participants had received education on the prevention of major infectious diseases. Almost all respondents had participated in COVID-19 knowledge training.

**Table 1:** Demographic characteristics of the survey participants.

**Variable**	**Participants (*n* = 774)**
**Age (years)**	
21–30	468 (60.47)
31–40	215 (27.78)
41–50	74 (9.56)
51 and above	17 (2.20)
**Gender**	
Male	19 (2.45)
Female	755 (97.55)
**Length of nursing service**, mean ± SD	9.30 ± 6.15
**Professional title**	
Nurse	236 (30.49)
Nurse practitioner	295 (38.11)
Nurse - in - charge	184 (23.77)
Associate professor of nursing	49 (6.33)
Professor of nursing	10 (1.29)
**Educational attainment**	
Junior college	265 (34.24)
Bachelor and above	509 (65.76)
**Marital status**	
Single	307 (39.66)
Married	458 (59.17)
Divorced	9 (1.16)
**Children**	
Zero	363 (46.90)
One	278 (35.92)
Two	133 (17.18)
**Children's education**	
Preschool education	297 (38.37)
Primary school	34 (4.39)
Junior high school	80 (10.34)
**Care experience^a^**	
Yes	62 (8.01)
No	712 (91.99)
**Protective education^b^**	
Yes	761 (98.32)
No	13 (1.68)
**COVID-19^c^ training**	
Yes	769 (99.35)
No	5 (0.65)
**Access to information**	
Mobile phone	750 (96.90)
TV-Computer	24 (3.10)

*Values are numbers (percentage) unless otherwise noted*.

*SD, standard deviation*.

a *Care experience such as SARS or influenza A H1N1*.

b *Protective education, received major infectious disease protection education for a disease such as SARS or influenza A H1N1*.

c *COVID-19, novel coronavirus disease*.

### Respondents' Ethical Cognition Toward COVID-19 Patients

A total of 427 participants (55.17%) believed that patients should be properly cared for, and affirmed the value of caring for patients, but were not willing to do so. The average score for the questionnaire about ethical cognition of patients' rights was 4.09 ± 0.59. Furthermore, 197 (25.45%) nurses believed that they had the right to refuse care of patients with infectious diseases, while 98 (12.66%) believed they had the right to refuse care even though the professional roles and responsibilities of nurses were emphasized, as shown in Table 2.

**Table 2:** Respondents' beliefs about the rights and interests while caring for COVID-19 patients (*n* = 774).

**Variable**	**Agree^a^**	**Disagree^b^**	**Neutral**	**Score**
COVID-19 patients have the right to expect the same quality of healthcare as other patients	722 (93.28)	14 (1.81)	38 (4.91)	4.36 ± 0.67
Nurses have the right to refuse care for COVID-19 patients	197 (25.45)	420 (54.26)	157 (20.28)	3.44 ± 1.13
Nurses do not have the right to refuse care for COVID-19 patients when professional roles and responsibilities are emphasized	569 (73.51)	98(12.66)	107 (13.82)	3.82 ± 0.97
Nurses cannot refuse to care for COVID-19 patients and should treat them equally	714 (92.25)	20 (2.58)	40 (5.17)	4.23 ± 0.68
Care is very important for patients with COVID-19	762 (98.45)	0	12 (1.55)	4.60 ± 0.52

*Values are numbers (percentage) unless otherwise noted*.

*Score: mean ± SD (standard deviation)*.

a *Agree: a response of “Agree” or “Strongly agree”*.

b *Disagree: a response of “Disagree” or “Strongly disagree”*.

*Scoring range: positive 4–5, negative: 1–2*.

### Respondents' Behavioral Intentions and Attitude Toward COVID-19 Patients

Overall, 69.12% (*n* = 535) of nurses reported a positive willingness to care for COVID-19 patients, with an average score of 3.95 ± 0.70 on the questionnaire. Furthermore, 83.33% of nurses were willing to cooperate with the health department to take care of COVID-19 patients and 69.77% were willing to take care of COVID-19 patients as a volunteer. In addition, 75.58% (*n* = 585) of participants had a positive attitude toward taking care of COVID-19 patients, with an average score of (4.00 ± 0.60), as shown in Table 3.

**Table 3:** Respondents' behavioral intentions and attitude toward caring for COVID-19 patients.

**Variable**	**Agree^a^**	**Disagree^b^**	**Neutral**	**Score**
**Behavioral intention**				
Assigned by the hospital to care for COVID-19 patients	619 (79.97)	24 (3.10)	131 (16.93)	3.97 ± 0.70
Volunteer to care for COVID-19 patients	540 (69.77)	42 (5.43)	192 (24.81)	3.84 ± 0.81
Cooperate with the dispatch of health administration department	645 (83.33)	18 (2.33)	111 (14.34)	4.03 ± 0.68
**Attitude**				
Caring for patients with COVID-19 is a good thing	463 (59.82)	69 (8.91)	242 (31.27)	3.66 ± 0.88
Caring for patients with COVID-19 is worth doing	632 (81.65)	16 (2.07)	126 (16.28)	4.09 ± 0.66
Caring for patients with COVID-19 is the right thing	651 (84.11)	22 (2.84)	101 (13.05)	4.06 ± 0.69
Caring for patients with COVID-19 is meaningful thing	613 (79.20)	28 (3.62)	133 (17.18)	4.19 ± 0.61

*Values are numbers (percentage) unless otherwise noted*.

*Score: mean ± SD (standard deviation)*.

a *Agree: a response of “Agree” or “Strongly agree”*.

b *Disagree: a response of “Disagree” or “Strongly disagree”*.

*Scoring range: positive 4–5, negative: 1–2*.

### Respondents' Normative Beliefs and Compliance Motivations for Caring for COVID-19 Patients

Nurses indicated the highest level of support from nursing professional associations (nursing associations) and hospital authorities (83.97%, *n* = 650), and the lowest levels of support from parents/relatives and friends, with 51.93% (*n* = 402) and 53.10% (*n* = 411), respectively. In terms of caring for patients, participants' compliance with the wishes of hospital directors was highest (*n* = 592, 76.49%), while compliance with family and friends was only 40.96% (*n* = 317) and 26.09% (*n* = 202), respectively, as shown in Supplementary Tables 1, 2.

### Respondents' Self-Efficacy for Caring for COVID-19 Patients

Of all respondents, 63.82% (*n* = 494) reported being confident about taking care of patients with COVID-19 with an average score of 3.94 ± 0.63 and a positive attitude. In the absence of special allowance and support from family and friends, the proportion of participants reporting positive self-efficacy was slightly lower than that for other items (67.83 and 70.41%), as shown in Table 4.

**Table 4:** Respondents' self-efficacy of caring for COVID-19 patients.

**Variable**	**Confidence^a^**	**Lack confidence^b^**	**Neutral**	**Score**
Family is worried	610 (78.81)	19 (2.45)	145 (18.73)	3.95 ± 0.70
Fear of infection	616 (79.59)	18 (2.33)	140 (18.09)	3.95 ± 0.69
Suffer strange looks	619 (79.97)	20 (2.58)	135 (17.44)	3.95 ± 0.70
Without subsidies	525 (67.83)	66 (8.53)	183 (23.64)	3.78 ± 0.75
Family and friends don not support	545 (70.41)	33 (4.26)	196 (25.32)	3.82 ± 0.77
Hassle to put on and take off protective gear	674 (87.08)	11 (1.42)	89 (11.50)	4.06 ± 0.62

*Values are numbers (percentage) unless otherwise noted*.

*Score: mean ± SD (standard deviation)*.

a *Confidence: a response of “confidence” or “strong confidence”*.

b *Lack confidence: a response of “lack confidence” or “strong lack of confidence”*.

*Scoring range: positive 4–5, negative: 1–2*.

### Factors Influencing Respondents' Behavioral Intentions to Care for COVID-19 Patients

Correlation analysis indicated a significant positive correlation between attitude, subjective normative beliefs, self-efficacy, nursing ethics cognition, and nursing behavioral intentions; self-efficacy and behavioral intentions had the highest correlation (r = 0.719, *P* < 0.001), followed by attitude, subjective norms, and nursing ethics cognition (r = 0.690, 0.603, and 0.546, respectively, all *P* < 0.001). These results indicated that the more positive a respondent's self-efficacy, attitude, subjective norms, and nursing ethics cognition related to COVID-19 patients, the higher their willingness to provide care to COVID-19 patients.

The stepwise analysis results showed that 63.3% of the variation in behavioral intentions could be explained by attitude, subjective norms, self-efficacy, and ethics cognition as independent variables (*F* = 331.02, *P* < 0.001, Table 5), while 51.6% of the variation could be explained by self-efficacy alone. The best standardized regression formula for prediction was: care intention = 0.260 × attitude + 0.056 × subjective norm + 0.083 × nursing ethical cognition + 0.214 × self-efficacy −1.081. Attitude and self-efficacy were the main predictors of behavioral intentions.

**Table 5:** Factors influencing respondents' behavioral intentions to care for COVID-19 patients.

**Dependent variable**	***B***	**SE**	**β**	***t***	***P*-value**
Constant	−1.081	0.384	–	−2.819	0.005
Attitude	0.260	0.027	0.300	9.513	<0.001
Subjective norm	0.186	0.012	0.137	4.670	<0.001
Ethical cognitive	0.083	0.017	0.389	12.584	<0.001
Self-efficacy	0.214	0.020	0.117	4.230	<0.001

### Structural Equation Modeling Results

**Figure 2:**
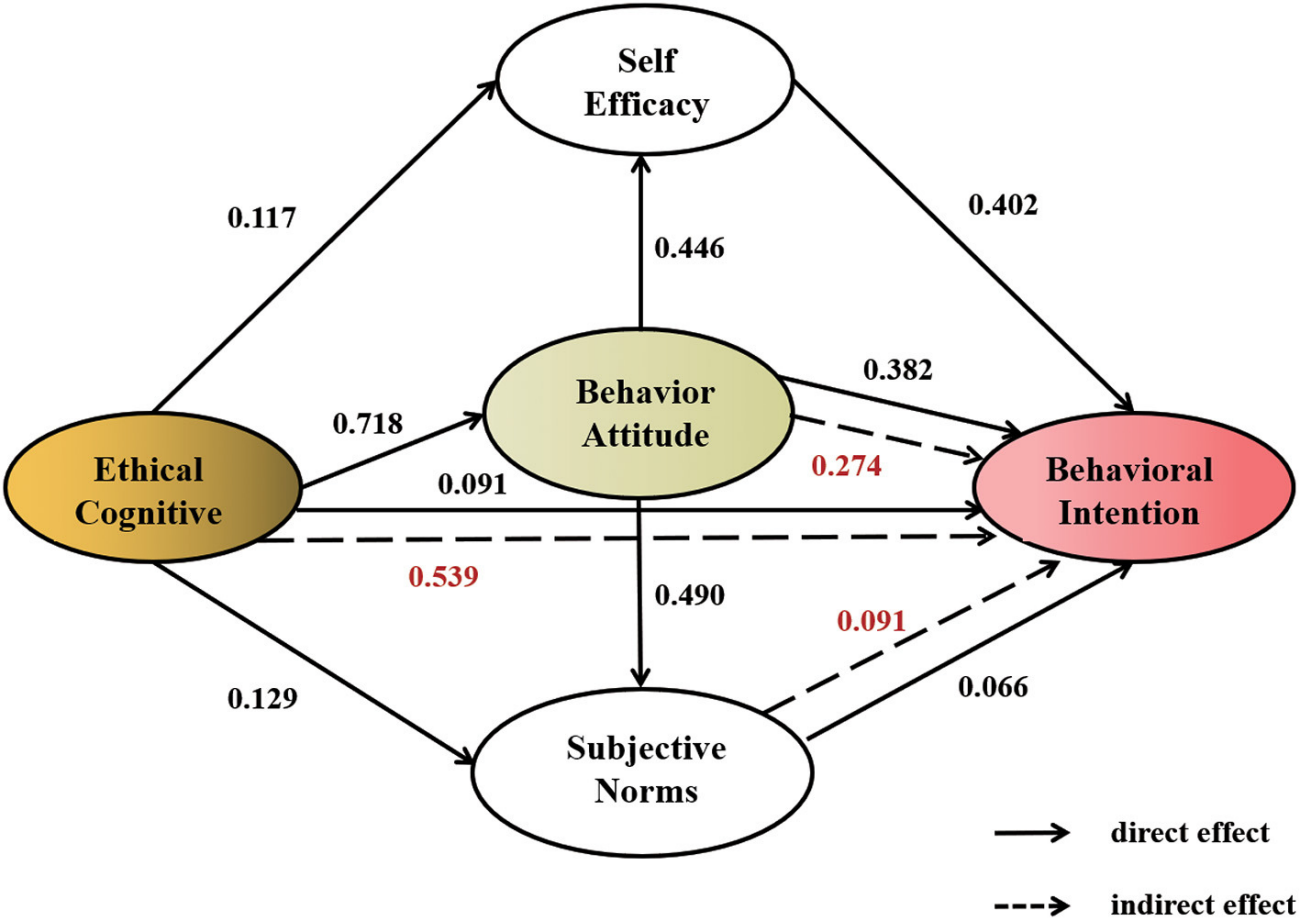
Influence pathway model of behavioral intentions to care for COVID-19 patients.

The relationship between the path coefficients and variables of the model framework of factors affecting nurses' behavioral intentions is shown in Figure 2. The construct reliability (NC) value of the absolute fit degree of this model was 2.82. The root mean square of approximate residual error (RMSEA) was 0.062 and the other fit degree indexes were all over 0.85 (the GFI, AGFI, NFI, CFI, IFI, and TLI values were 0.887, 0.866, 0.933, 0.943, 0.943, and 0.937, respectively). These results indicate a good degree of fit and that the model could explain the observed variables and potential variables as well as the relationship between variables, as shown in Supplementary Table 3.

The standardized path coefficients of cognition on attitude, self-efficacy, and subjective norms were 0.718, 0.117, and 0.129, respectively (all *P* < 0.01, Figure 2). The standardized path coefficients of cognition, subjective norms, attitude, and self-efficacy on behavioral intentions were 0.091, 0.066, 0.382, and 0.402, respectively, and statistically significant (*P* < 0.01, Figure 2). These results indicate that cognition, attitude, subjective norms, and self-efficacy had direct positive effects on behavioral intentions. The influence of attitude, cognition, self-efficacy, and subjective norms on the total effect of behavioral intentions was 0.656, 0.630, 0.402, and 0.157, respectively (*P* < 0.01, Table 6). Among them, self-efficacy only had a direct effect on behavioral intention, and its influence on behavioral intentions was the largest. The direct effect of cognition on behavioral intention was 0.091, while the indirect effect was 0.539; thus, the indirect effect on behavioral intentions was larger (Table 6).

**Table 6:** Effects of attitude, cognition, self-efficacy, and subjective norms on behavioral intentions.

**Variable**	**Behavioral intentions**		
	**Direct effect**	**Indirect effect**	**Total effect**
Self-efficacy	0.402^***^ (14.993)	0.000	0.402
Behavioral attitude	0.382^***^ (10.758)	0.274	0.656
Cognitive	0.091^**^ (3.072)	0.539	0.630
Subjective norms	0.066^**^ (3.019)	0.091	0.157

*** *P < 0.001*;

** *P < 0.01; () represents the corresponding value of construct reliability*.

## Discussion

The COVID-19 pandemic has highlighted many of the ethical challenges that healthcare professionals confront while caring for patients and their families (17). As the largest global healthcare workforce, nurses' perspectives are not always considered in the face of sudden outbreaks. A nurse's primary duty is to care for patients; however, in the context of the COVID-19 pandemic, some of the necessary steps to protect the public have created new and unfamiliar tensions between nurses and patients and their families. Khatatbeh et al. (10) found that Jordanian HCWs feared infection and worried about their families, but they are still willing to join the battle, took up their responsibilities, concentrated on their duties, and showed professional commitment. In addition to work-related challenges, participants were challenged by their attachment to their family and being the source of infection.

Our results indicated that 55.17% of nurses were hesitant and ambivalent about their willingness to care for infectious patients in person. Although 93.28% of nurses believed that COVID-19 patients had the right to expect the same quality of healthcare as other patients, 25.45% were not willing to care for such patients themselves, resulting in significant ethical challenges. Possible reasons for these findings include pathophysiology, mode of transmission, susceptibility, and infectivity characteristics, as well as failures in the supply chain to provide healthcare workers with the personal protective equipment necessary for them to take on substantial but uncertain risk (17). Most of the health care workers reported that they were challenged with facing social discrimination as a result of working with COVID-19 (10). As professionals, nurses acknowledge the value and significance of caring for patients, but are also concerned about spreading COVID-19 to loved ones—especially children or dependent adult relatives that they are the sole supporter and caregiver for (18). Kim et al. (19) found that nurses felt conflicted between their professional responsibilities to care for SARS patients and their personal safety. This is consistent with the American Nurses Association Code of Ethics and the Code of Professional Ethics (20).

The results indicated that ethics cognition had a small direct effect on behavioral intention as well as a significant positive effect on attitude and self-efficacy, attitude and self-efficacy had a significant positive effect on behavioral intentions, and cognition had a partial mediating effect on behavioral intentions. Cognition indirectly affects behavioral intentions through the mediating effect of attitude and self-efficacy. Therefore, it is necessary for nursing managers to encourage discussion of nursing ethics cognition, especially during the outbreak of infectious diseases.

The results of our survey showed that 69.12% of nurses were willing to care for patients with COVID-19 and 75.58% had a positive attitude. This may be related to the professional responsibilities and ethical cognition of nurses (21). Both the multiple regression analysis and structural equation model results indicated that attitude had the greatest influence on behavioral intentions, indicating that the more positive the behavioral attitude, the stronger the behavioral intentions. A study by Kim et al. (19) found that nurses' negative reactions were not due to lack of knowledge or ignorance and unwillingness to care for SARS patients, but a negative attitude or low sense of self-efficacy. When negative beliefs are ignored they may affect nursing behaviors, thereby reducing the quality of care. Andersson et al. (22) found that behavioral attitudes are predictors of willingness to care, and that when a person expects a positive outcome from their actions, their attitude toward that behavior is also positive. Khalid et al. (23) reported that positive feedback and economic benefits from colleagues could reduce nurses' fear and pressure and enhance their willingness to work during epidemic outbreaks. Nashwan et al. (9) found that nurses are more willing to work with COVID-19 patients when they are more knowledgeable and well-compensated for the level of work-environment-related risks. Therefore, nursing managers should praise and encourage nurses while striving to provide additional subsidies to change nurses' attitudes and increase their willingness to care.

The correlation analysis results in the present study showed significant positive correlations of attitude with subjective norms, self-efficacy, ethics cognition, and behavioral intention, with self-efficacy having the strongest correlation. Furthermore, the multiple linear regression analysis and SEM results showed that self-efficacy had the greatest direct influence on behavioral intentions with a direct effect value of 0.402, indicating that self-efficacy has a direct positive prediction effect on behavioral intentions, i.e., the higher the level of self-efficacy, the stronger the behavioral intention. This is consistent with the conclusion of Kim et al. (19) that clinical nurses with a more positive attitude, stronger self-efficacy, and greater approval from their significant others were more likely to have stronger intentions to care for SARS patients. A systematic review and meta-analysis showed that confidence in safety, risk perception, prior training, general and role knowledge and confidence in skills were proven facilitators for willingness to work during an influenza pandemic (24).

Subjective norms had the least influence on behavioral intentions. This finding is consistent with that of Lee et al. (8), who found that subjective norms have a weaker influence on willingness than attitude and perceived behavioral control. However, this finding is inconsistent with Connor (25) and Minuye et al. (11), whose studies indicated that subjective norms have a large influence on intention. These inconsistencies could be due to the definition of subjective norms and differences between the study samples, environments, and behaviors that impact subjective norms. Khatatbeh et al. (10) found that the families, colleagues, organizations, self, and community's support play a significant role in motivating the HCWs during the pandemic. The results of our survey also revealed that parents/relatives are the main group having a negative influence on nurses' behavioral intentions to care for COVID-19 patients. However, due to their sense of professional responsibility, nurses did not report high compliance and hold a neutral attitude to parents/relatives' will, which may be the reason for their small influence on behavioral intentions. This is consistent with the conclusion of Khatatbeh et al. (10) that COVID-19 pandemic is a special situation and professionally they must be responsible, and it is their duty.

## Limitations

Although the findings of this study have implications for exploring factors influencing nurses' willingness to care for COVID-19 patients, there are some limitations. First, this study investigated behavioral intention toward caring for COVID-19 patients rather than actual caring behavior. Compared with actual behavior, behavioral intention may be overestimated. Second, the study's cross-sectional design is a limitation where correlation and not causation could be inferred. Third, Self-reported data bias was inevitable, the small sample size and convenience sample may have resulted in selection bias, cannot fully reflect the current behavioral intentions of all clinical nurses to care for patients with infectious diseases. Fourth, the sample was primarily drawn from the southeast region of China, limiting the generalizability of the findings to the rest of the country. Longitudinal study was needed to investigate the predictors of behavioral intention among frontline nurses in the future.

## Conclusions

The results of this study revealed that an integrated model based on self-efficacy and behavioral planning theory can better explain and predict the behavioral intention of clinical nurses to care for patients with infectious diseases. To improve nurses' behavioral intentions, nursing managers should focus on strengthening positive attitudes toward patients with infectious diseases, improving the level of self-efficacy, and providing training to teach relevant knowledge.

## Data Availability Statement

The original contributions presented in the study are included in the article/Supplementary Material, further inquiries can be directed to the corresponding author/s.

## Ethics Statement

Written informed consent was obtained from the individual(s) for the publication of any potentially identifiable images or data included in this article.

## Author Contributions

FC, YuJ, HW, and JX were involved in conception and study design. They were responsible for data collection together with RW, SC, HF, and YaJ. FC was involved in writing the manuscript and was responsible for the data analysis. XP and MY were involved in revising the manuscript. All authors were responsible for critical revision of the manuscript. All authors contributed to the article and approved the submitted version.

## Conflict of Interest

The authors declare that the research was conducted in the absence of any commercial or financial relationships that could be construed as a potential conflict of interest.

## Publisher's Note

All claims expressed in this article are solely those of the authors and do not necessarily represent those of their affiliated organizations, or those of the publisher, the editors and the reviewers. Any product that may be evaluated in this article, or claim that may be made by its manufacturer, is not guaranteed or endorsed by the publisher.
