# Supplementary material for: Behavioral Intentions and Factors Influencing Nurses' Care of COVID-19 Patients: A Cross-Sectional Study
Source: Front Public Health. 2022 Jun 29;10:914599. doi: 10.3389/fpubh.2022.914599 (PMC9278515; doi:10.3389/fpubh.2022.914599)
Supplement: Supplementary file 1 [file Table_1.DOCX]

Supplementary Material

## Supplementary Tables

**Supplementary Table 1.** Influences of significant others or groups on respondents' behavioral decisions about caring for COVID-19 patients (n = 774).

| **Variable** | **Support** | **Nonsupport** | **Neutral** | **Score** |
| --- | --- | --- | --- | --- |
| Parents/relatives | 402 (51.93) | 118 (15.25) | 254 (32.82) | 3.43 ± 0.92 |
| Friends | 411 (53.10) | 76 (9.82) | 287 (37.08) | 3.51 ± 0.82 |
| General public (non-medical staff) | 641 (82.82) | 14 (1.81) | 119 (15.37) | 4.06 ± 0.70 |
| Section head or head nurse | 640 (82.69) | 3 (0.39) | 131 (16.93) | 4.07 ± 0.66 |
| Nursing professional association | 650 (83.98) | 2 (0.26) | 122 (15.76) | 4.11 ± 0.66 |
| Hospital director | 650 (83.98) | 3 (0.39) | 121 (15.63) | 4.11 ± 0.67 |
| Unit nursing colleagues | 640 (82.69) | 2 (0.26) | 132 (17.05) | 4.09 ± 0.67 |
| Department of health | 645 (83.33) | 1 (0.13) | 128 (16.54) | 4.11 ± 0.67 |
| Patients and Family members | 632 (81.65) | 6 (0.78) | 136 (17.57) | 4.08 ± 0.69 |

Values are numbers (percentage) unless otherwise noted.

Score: mean ± SD (standard deviation).

Support: includes support and strong support.

Nonsupport: includes nonsupport and strong nonsupport.

Scoring range: positive 4–5, negative: 1–2.

**Supplementary Table 2.** Participants’ willingness to comply with important reference objects while caring for COVID-19 patients.

| **Variable** | **Compliance** | **Noncompliance** | **Neutral** | **Score** |
| --- | --- | --- | --- | --- |
| Parents/relatives | 317 (40.96) | 72 (9.30) | 385 (49.74) | 3.36 ± 0.72 |
| Friends | 202 (26.09) | 175 (22.61) | 397 (51.30) | 3.06 ± 0.78 |
| General public (non-medical staff) | 190 (24.55) | 187 (24.16) | 397 (51.29) | 3.02 ± 0.79 |
| Section head or head nurse | 571 (73.77) | 19 (2.45) | 184 (23.77) | 3.79 ± 0.63 |
| Nursing professional association | 581 (75.06) | 13 (1.68) | 180 (23.26) | 3.83 ± 0.62 |
| Hospital director | 592 (76.49) | 10 (1.29) | 172 (22.22) | 3.85 ± 0.59 |
| Unit nursing colleagues | 508 (65.63) | 19 (2.45) | 247 (31.91) | 3.70 ± 0.65 |
| Department of health | 589 (76.10) | 9 (1.16) | 176 (22.74) | 3.83 ± 0.59 |
| Patients and family members | 444 (57.36) | 28 (3.62) | 136 (17.57) | 3.59 ± 0.67 |

Values are numbers (percentage) unless otherwise noted.

Score: mean ± SD (standard deviation).

Support: includes support and strong support.

Nonsupport: includes nonsupport and strong nonsupport.

Scoring range: positive 4–5, negative: 1–2.

**Supplementary Table 3.** Significance test of load coefficient/path coefficients of the modified model.

| **Latent variable** | **Item** | **Unstandardized estimates** | **Standardized estimates** | ***P*** | **Reliability coefficient** | **Measurement error** | **CR** | **AVE** | **Cronbach’s α** |
| --- | --- | --- | --- | --- | --- | --- | --- | --- | --- |
| Ethical cognitive | Item 1 | 1.000 | 0.553 | - | 0.306 | 0.694 | 0.799 | 0.523 | 0.757 |
|  | Item 2 | 1.893 | 0.663 | ** | 0.440 | 0.560 |  |  |  |
|  | Item 3 | 1.730 | 0.701 | ** | 0.491 | 0.509 |  |  |  |
|  | Item 4 | 1.434 | 0.834 | ** | 0.696 | 0.304 |  |  |  |
|  | Item 5 | 0.787 | 0.558 | ** | 0.311 | 0.689 |  |  |  |
| Behavioral intentions | Item 6 | 1.000 | 0.961 | - | 0.924 | 0.076 | 0.958 | 0.883 | 0.946 |
|  | Item 7 | 1.060 | 0.926 | ** | 0.857 | 0.143 |  |  |  |
|  | Item 8 | 0.925 | 0.931 | ** | 0.867 | 0.133 |  |  |  |
| Behavioral attitude | Item 9 | 1.000 | 0.700 | - | 0.490 | 0.510 | 0.897 | 0.688 | 0.856 |
|  | Item 10 | 0.958 | 0.877 | ** | 0.769 | 0.231 |  |  |  |
|  | Item 11 | 0.995 | 0.869 | ** | 0.755 | 0.245 |  |  |  |
|  | Item 12 | 0.877 | 0.858 | ** | 0.736 | 0.264 |  |  |  |
| Normative beliefs | Item 13 | 1.000 | 0.938 | - | 0.880 | 0.120 | 0.908 | 0.640 | 0.878 |
|  | Item 14 | 1.023 | 0.954 | ** | 0.910 | 0.090 |  |  |  |
|  | Item 15 | 0.956 | 0.918 | ** | 0.843 | 0.157 |  |  |  |
|  | Item 16 | 0.920 | 0.872 | ** | 0.760 | 0.240 |  |  |  |
|  | Item 17 | 0.543 | 0.493 | ** | 0.243 | 0.757 |  |  |  |
|  | Item 18 | 0.684 | 0.452 | ** | 0.204 | 0.796 |  |  |  |
| Compliance motivation | Item 19 | 1.000 | 0.410 | - | 0.168 | 0.832 | 0.888 | 0.592 | 0.823 |
|  | Item 20 | 1.368 | 0.430 | ** | 0.185 | 0.815 |  |  |  |
|  | Item 21 | 2.751 | 0.890 | ** | 0.792 | 0.208 |  |  |  |
|  | Item 22 | 2.779 | 0.926 | ** | 0.857 | 0.143 |  |  |  |
|  | Item 23 | 2.736 | 0.934 | ** | 0.872 | 0.128 |  |  |  |
|  | Item 24 | 2.619 | 0.822 | ** | 0.676 | 0.324 |  |  |  |
| Self-efficacy | Item 25 | 1.000 | 0.899 | - | 0.808 | 0.192 | 0.955 | 0.781 | 0.949 |
|  | Item 26 | 1.045 | 0.919 | ** | 0.845 | 0.155 |  |  |  |
|  | Item 27 | 1.019 | 0.916 | ** | 0.839 | 0.161 |  |  |  |
|  | Item 28 | 0.989 | 0.822 | ** | 0.676 | 0.324 |  |  |  |
|  | Item 29 | 1.071 | 0.864 | ** | 0.746 | 0.254 |  |  |  |
|  | Item 30 | 0.895 | 0.878 | ** | 0.771 | 0.229 |  |  |  |
| Subjective norms | Item 31 | 1.000 | 0.856 | - | 0.733 | 0.267 | 0.875 | 0.573 | 0.726 |
|  | Item 32 | 1.786 | 0.824 | ** | 0.524 | 0.476 |  |  |  |
|  | Item 33 | 1.854 | 0.867 | ** | 0.572 | 0.428 |  |  |  |
|  | Item 34 | 2.143 | 0.852 | ** | 0.726 | 0.274 |  |  |  |

** *P* < 0.001
